# The Dynamic Right-to-Left Translocation of Cerl2 Is Involved in the Regulation and Termination of Nodal Activity in the Mouse Node

**DOI:** 10.1371/journal.pone.0060406

**Published:** 2013-03-27

**Authors:** José Manuel Inácio, Sara Marques, Tetsuya Nakamura, Kyosuke Shinohara, Chikara Meno, Hiroshi Hamada, José António Belo

**Affiliations:** 1 Regenerative Medicine Program, Departamento de Ciências Biomédicas e Medicina, Universidade do Algarve, Faro, Portugal; 2 IBB-Institute for Biotechnology and Bioengineering, Centro de Biomedicina Molecular e Estrutural, Universidade do Algarve, Campus de Gambelas, Faro, Portugal; 3 Developmental Genetics Group, Graduate School of Frontier Biosciences, Osaka University, and Core Research for Evolutional Science and Technology, Japan Science and Technology Corporation, Osaka, Japan; 4 Graduate School of Medical Sciences, Dept Dev Biol, Kyushu University, Fukuoka, Japan; New York Medical College, United States of America

## Abstract

The determination of left-right body asymmetry in mouse embryos depends on the interplay of molecules in a highly sensitive structure, the node. Here, we show that the localization of Cerl2 protein does not correlate to its mRNA expression pattern, from 3-somite stage onwards. Instead, Cerl2 protein displays a nodal flow-dependent dynamic behavior that controls the activity of Nodal in the node, and the transmission of the laterality information to the left lateral plate mesoderm (LPM). Our results indicate that Cerl2 initially localizes and prevents the activation of Nodal genetic circuitry on the right side of the embryo, and later its right-to-left translocation shutdowns Nodal activity in the node. The consequent prolonged Nodal activity in the node by the absence of Cerl2 affects local *Nodal* expression and prolongs its expression in the LPM. Simultaneous genetic removal of both Nodal node inhibitors, Cerl2 and Lefty1, sustains even longer and bilateral this LPM expression.

## Introduction

During embryonic development, the establishment of the Left-Right (L-R) axis is critical for the proper asymmetric positioning of the internal organs, and correct development of the forming organs itself [1]–[3]. Loss of asymmetry is associated to several human diseases, namely heterotaxia syndrome, congenital heart disease, or primary ciliary dyskinesia [4], [5].

The initial event in symmetry-breaking proceeding is the asymmetric generation of a signal, in the mouse node at embryonic day (E) 7.5, that is transferred preferentially towards the left side of the lateral plate mesoderm (LPM; reviewed in [1], [6]). Nodal is a secreted protein, a member of the transforming growth factor-beta (TGF-β) superfamily, that plays a crucial role in L-R patterning [7], [8]. Although not fully understood, it is proposed that the up-regulation of the expression of *Nodal* in the cells on the left side of the perinodal region, prompts its expression in the left-LPM as well as the induction of *Nodal* downstream genes [9]–[11]. Nevertheless, the leftward fluid flow generated by the cilia localized in the node of the mouse embryo has been considered essential and sufficient for L-R asymmetry establishment [6]. It is yet not clear, however, how the flow influences the signals involved in the breaking of L-R symmetry. The two principal hypotheses prompt for a flow-transported determinant molecule or a flow-generated mechanical stress sensed by the node cells [for a review, see [12]].

Cerl2 is a secreted 20-kDa protein belonging to the family of TGF-β antagonists, Cerberus/DAN [13], whose gene transcripts can be detected in the perinodal region at the early headfold (EHF) stage of mouse embryo development [14]. In theory, Cerl2 summons the prime properties to answer symmetry breaking, it is a protein with the hypothetical size to create a stationary accumulation on the left side of the node due to the laminar flow [15]. Moreover, *Cerl2* knockout mice show a wide range of laterality defects including randomization of *Nodal* expression in the LPM, which substantiates the involvement of Cerl2 in the specification of the mouse L-R axis [14].

Here, we demonstrate the extracellular nature of Cerl2 protein and its dynamic localization on the node of mouse embryos. The results show an accumulation of Cerl2 protein on the right side of the node at 2/3-somite stage that deviates to the left side at 4/5-somite stage. Moreover, we clearly show that this behavior of Cerl2 is nodal flow dependent. In addition, we observed that in the absence of Nodal antagonism in the node, by loss of both Cerl2 and Lefty1, the expression of *Nodal* in LPM became always bilateral, earlier and wider than expected. Our results demonstrate that the maintenance of the correct levels of *Nodal* in the node are crucial for proper L-R axis establishment, and that Cerl2 activity is essential first to prevent the activity of Nodal in the right-LPM and later to shutdown Nodal activity in the left side of the node and consequently in the left-LPM, in a precise time window.

## Materials and Methods

### Ethics Statement

The studies involving animal experiments are in accordance to the ethical issues for clinical research and EU guidelines for animal research. All animal work performed in this study was conducted compliant with the Portuguese law and approved by the Consultive Commission of the Veterinary Agency (Portuguese Ministry of Agriculture), the sole Agency/Committee in Portugal responsible to issue the ethical approval for these type of studies, following the EU guidelines for animal research and welfare.

### Mice

Briefly, mice were maintained on a 7 pm to 5 am dark cycle and mated overnight. Mouse embryos were obtained by crossing mice and embryonic development was staged according to gestational age, with noon of the day of vaginal plug detection being considered E0.5. Pregnant females were sacrificed by cervical dislocation and the uteri were surgically removed and placed in ice-cold PBS. Embryos were dissected out of the *decidua* with fine forceps and staged according to morphological landmarks.

The *Cerl2^(−/−)^* and *Lefty1^(+/−)^* mice lines were previously described [14], [16]. To establish a stable *Cerl2^(−/−)^; Lefty1^(+/−)^* mice line, the heterozygous *Lefty1^(+/−)^* mice were mated with the homozygous *Cerl2^(−/−)^*. These animals, *Cerl2^(−/−)^*; *Lefty1^(+/−)^*, were intercrossed in order to obtain *Cerl2^(−/−)^*; *Lefty1^(−/−)^* double-mutant embryos, and both mice and embryos were genotyped by the polymerase chain reaction. The embryos analyses were performed on a mixed C57Bl/129 background.

### Embryo culture and whole-mount immunofluorescence analysis

Embryos were collected at early headfold stage (EHF), and cultured in a rotating 50-ml Falcon tube under 5% CO2, 75% rat serum and 25% DMEM (Invitrogen) until reaching desired stage and fixed at 4°C with 4% paraformaldehyde.

Alternatively, embryonic day 7.5 to 8.5 mouse embryos were recovered in cold phosphate-buffered saline (PBS), and fixed as above. After permeabilization with 0.1% Triton X-100 in PBS, they were incubated with 2% BSA, 0.1% Triton X-100 in PBS, before exposure to goat polyclonal antibodies to Cerl2 (R&D) and rabbit polyclonal antibodies to laminin (when applied) at a dilution of 1 200 or 1 1000, respectively. Immune complexes were detected with AlexaFluor-conjugated secondary antibodies (Molecular Probes), and nucleus was stained with 4′,6-diamidino-2-phenylindole (DAPI). Embryos mounted onto glass slides using Mowiol mounting reagent and coverslipped. Images were taken using inverted LSM710 laser scanning confocal microscope (Zeiss).

### Whole-mount in situ hybridization

Whole-mount *in situ* hybridization and antisense probe preparation were performed according to standard procedures as described previously [17]. Details of the RNA probes provided upon request. Detection of *Uncx4.1* expression allowed accurate determination of the number of somite pairs (this probe was gently provided by Prof. Ahmed Mansouri, Max-Plank Institute).

### Cell transfections and Western blotting

Plasmid for expression of Cerl2-Flag was previously described [14]. Human embryonic kidney 293T cells were transiently transfected using lipofectamine in OptiMEM I reduced-serum medium (Invitrogen). Cell lysates and conditioned media were collected after 48 h, and protein expression was monitored by Western blotting using monoclonal M2 anti-Flag (Sigma), goat polyclonal anti-Cerl2 (R&D), and HRP-conjugated anti-mouse and anti-goat (Sigma) antibodies. Proteins were visualized using ECL detection reagent (Pierce).

Cerl2 and Nodal activities were monitored using a luciferase assay. Mixtures containing 0.5 µg of each Nodal [14], Cripto [18], Cerl2 [14], 50 ng of the luciferase reporter plasmid, 20 ng of CMV-β-Gal plasmid, and various amounts of pCS2+ vector to maintain a constant amount of total DNA were used to transfect 293T cells. Luciferase activity was measured 48 h after transfection, and the activities were normalized to β-galactosidase control.

### Mathematical Simulation

The mathematical model was constructed based on the SELI system with the use of MATHEMATICA software (Wolfram Media). The differential equations and additional information are presented in Nakamura *et al.*, 2006.

## Results and Discussion

### The localization of secreted Cerl2 protein does not correlate to its mRNA expression pattern, at 3-somite stage

**Figure 1:**
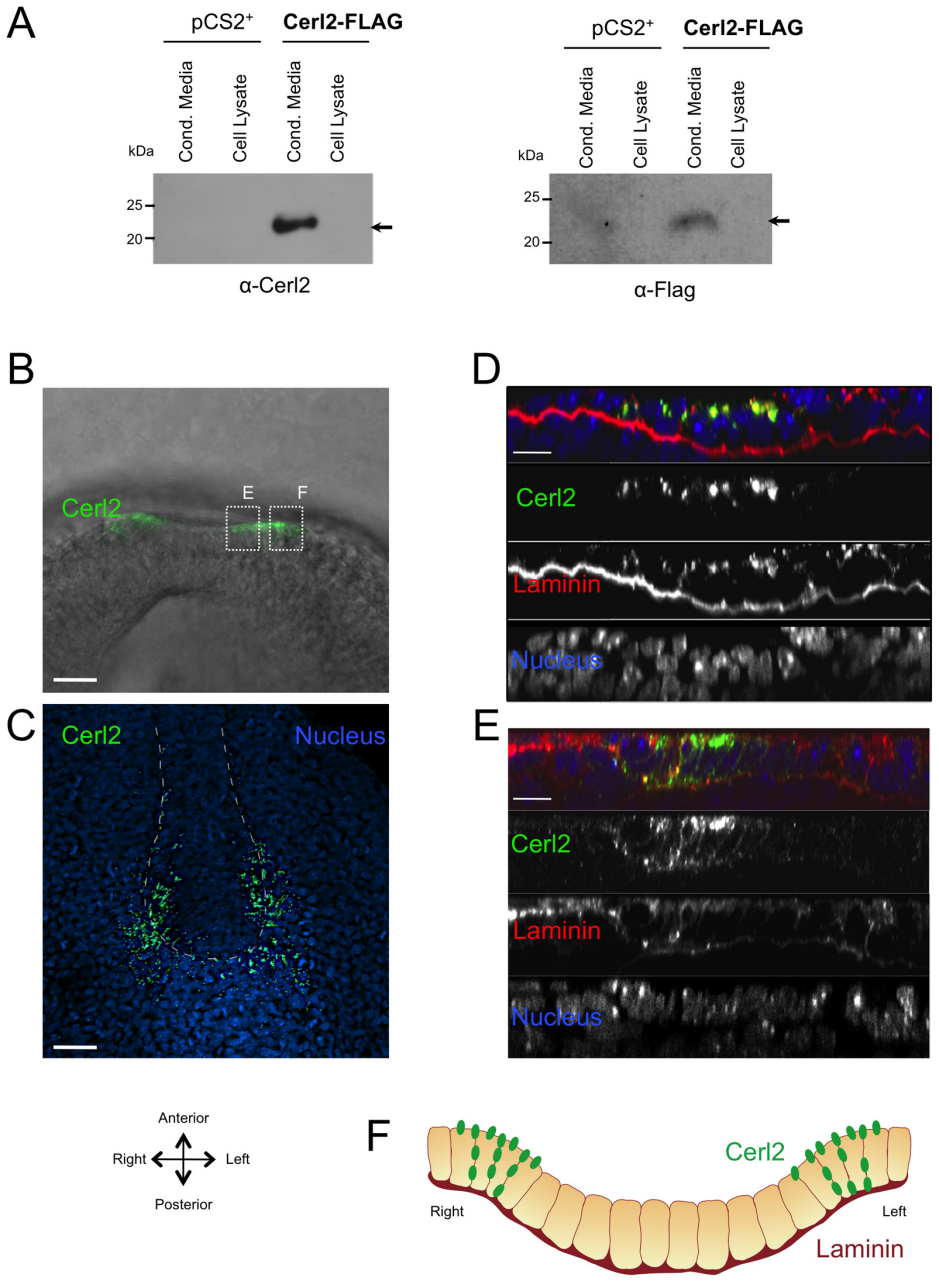
Immunofluorescence detection of Cerl2 at the node. (A) Western blot analysis of Cerl2-Flag in cell lysates and conditioned media of transfected 293T cells. The proteins were detected with anti-Flag, and anti-Cerl2 antibodies. The presence of the recombinant Cerl2 protein in the conditioned media confirmed its extracellular nature (indicated with an arrow). The size, in kilo Dalton, of the broad range molecular weight standards is indicated. (B) An E8.0 (3-somite stage) mouse embryo subjected to whole-mount immunofluorescence analysis with antibodies to Cerl2. (C) 3D reconstruction of confocal sections through the entire thickness of the node of a E8.0 mouse embryo stained to detect Cerl2 protein (green) and nuclei (DAPI, blue). (D and E) Orthogonal view through the node shows that Cerl2 localizes at the apical surface and lateral membrane. (F) Schematic transverse section of an E8.0 mouse embryo showing the localization of Cerl2 protein at the apical surface and lateral membrane of the perinodal crown cells.

**Figure 3:**
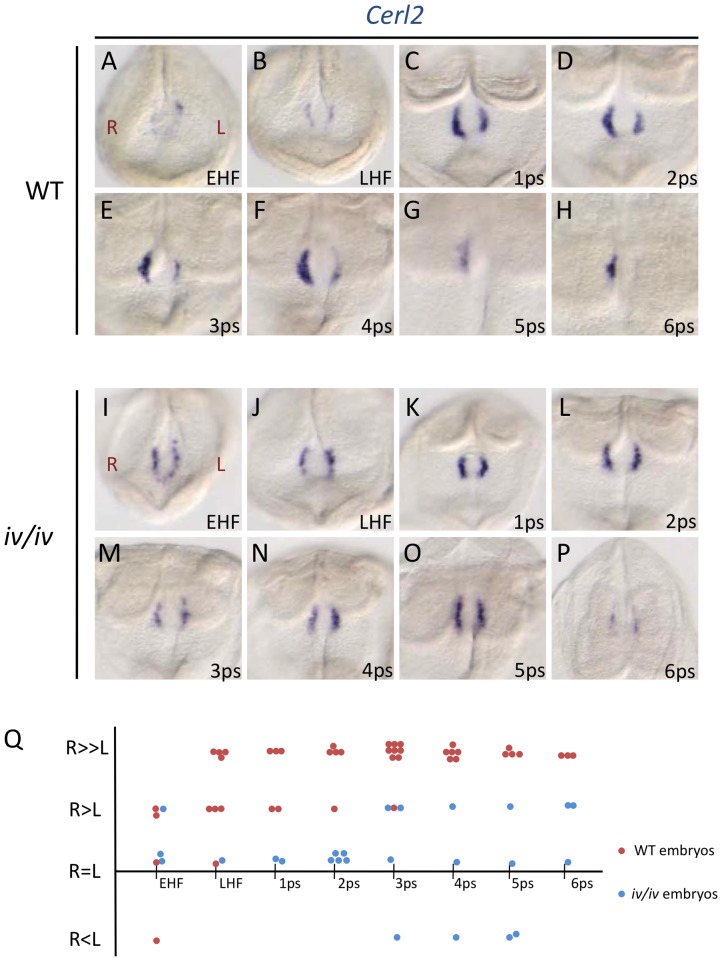
Time course and pattern of *Cerl2* RNA expression during early somitogenesis. Representative patterns of *Cerl2* RNA expression in the node of wild-type (A–H), and *iv/iv* (I–P) embryos at different developmental stages. (Q) Difference in the level of *Cerl2* RNA expression between the left and right sides of embryos at different developmental stages of the indicated genotypes: wild-type-red circles; *iv/iv*-blue circles. Each circle represents one embryo.

In order to access the subcellular localization of Cerl2 protein, we transfected 293T cells with a plasmid harboring the full-length of *Cerl2* coding region with a FLAG epitope tag inserted at C-terminus. The recombinant, Cerl2-FLAG, protein was successfully produced, and detected by Western blot at high levels in the conditioned media of transfected cells using either a Cerl2 antisera or an anti-FLAG antibody, evidencing that Cerl2 is an extracellular protein (Fig. 1A). Moreover, recombinant Cerl2 displays only one band with the molecular mass of 22 kDa (Fig. 1A). Interestingly, Okada and co-workers determined that only proteinaceous morphogens, secreted into the cavity of the ventral node, with a size of 20–40 kDa will be capable to generate a flow-dependent stationary gradient and eventually accumulate on the left side of the mouse node [15]. According to this, the size of Cerl2 protein prompts it as a secreted factor that might be distress by the leftward flow. Therefore, to examine the localization of Cerl2 protein in the mouse embryo, we performed whole-mount immunofluorescence (WIF) assays in embryos at 3-somite stage. The WIF was performed using the Cerl2 antisera utilized in the blotting, which was highly specific towards Cerl2 protein, and antibodies against laminin, which helped the visualization of the extracellular matrix, specially, the basement membrane [19]. The results showed that Cerl2 protein localizes in the perinodal crown cells of the node (Fig. 1B,C). In *Cerl2* KO embryos, no signal was detected (data not shown). In a more detailed analysis, we observe that the accumulation of Cerl2 is preferentially detected on the apical region of these cells, but a less intense signal was also detected in the basolateral region (Fig. 1D–F). A similar subcellular localization was observed for the 3XMyc-Nodal protein [11], indicating that both these molecules are detected in the same regions, immediately external to the apical and basolateral membranes of the perinodal cells. These observations suggest that, upon secretion, Cerl2 co-localizes with Nodal, what is not surprising since Cerl2 has been shown to bind to Nodal [14]. Curiously, however, was the even distribution of Cerl2 protein in both sides of the node at the 3-somite stage (Fig. 1B,C). At this stage, *Cerl2* gene displays an asymmetric expression on the right side of the mouse node (Fig. 3E) [14]. This means that, at 3-somite stage, the localization of Cerl2 protein does not correlate to its mRNA expression pattern.

### Cerl2 protein displays a nodal flow-dependent right-to-left translocation

**Figure 2:**
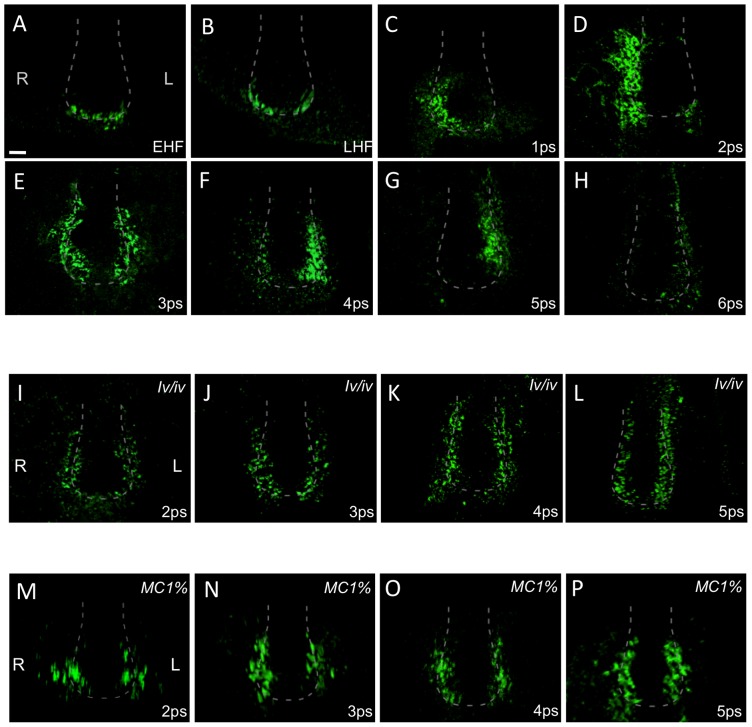
Distribution of Cerl2 at the node during early somitogenesis is nodal flow-dependent. Immunofluorescence detection of Cerl2 protein at the node during the indicated developmental stages in wild-type (A–H), *iv/iv* embryos (I–L), and wild-type embryos cultured in the presence of 1% methylcellulose (from EHF until the stage presented; M–P). A dashed line denotes the node region of the embryos. *n*≥5/5 embryos per stage were analyzed, except M-(3/3), N-(3/3), O-(2/2).

To get further information about the *in vivo* role of Cerl2, we decided to verify by WIF the localization of this protein throughout early somitogenesis. We observed that until the 2-somite stage, the localization of Cerl2 protein resembles the expression pattern of *Cerl2* transcripts (Fig. 2A-D and 3A-D). At these early stages, the protein was first detected in the perinodal crown cells on both sides of the node (EHF to LHF stage), in a horseshoe shape. Later, Cerl2 protein started to accumulate on the right side until 2-somite stage (Fig. 2D). Surprisingly, a signal transposition to the left side of the node was observed at 3- to 4-somite stages (Fig. 2E,F). At 5-somite stage, Cerl2 localized preferentially on the left side of the node, and the signal disappeared after 6-somite stage (Fig. 2G,H). Since Cerl2 protein matches the size of secreted morphogens that could be transported by the fluid flow (20–40 kDa), we analyzed if the translocation of Cerl2 from the right to the left side of the node was nodal flow-dependent. Hence, we performed a time-course experiment in *iv/iv* embryos, in which the flow is disabled due to immotile cilia [20]. The results showed that, in these mutants, the Cerl2 protein signal was always present in both sides of the node, and from early head-fold to 6-somite stages (Fig. 2I–L). It is noteworthy to mention that the Cerl2 protein localization and the *Cerl2* RNA expression pattern are quite similar in these *iv/iv* embryos (Fig. 3I–P).

Moreover, the effect of the leftward flow on the localization of Cerl2 protein was confirmed by the examination of embryos cultured in the presence of 1% methylcellulose, which mechanically abolishes the ciliary motion and fluid flow [21]. The embryos were cultured from EHF until 2-, 3-, 4-, and 5-somite stages, and the results showed a balanced distribution of Cerl2 protein on both sides of the node throughout early somitogenesis. The protein location at the node did not show right-sided bias at earlier somite stages, nor left-sided preponderance from 3-somite stage on (Fig. 2M–P).

All these observations strongly suggest that the right-to-left translocation of Cerl2 in the node is nodal flow-dependent, and that this transition starts right after the flow velocity becomes maximal, at 2-somite stage [21]. Interestingly, not only the localization of Cerl2 protein is nodal flow-dependent but also the expression of *Cerl2* in the node is regulated by a nodal flow-dependent mechanism. It has been demonstrated that, soon after the flow is generated (LHF stage) the expression of *Cerl2* in the perinodal region becomes asymmetric [21], but remains symmetric in the presence of an impaired flow [22], [23].

### The dynamic behavior of Cerl2 protein in the node is responsible for the time window expression of Nodal in the LPM

**Figure 4:**
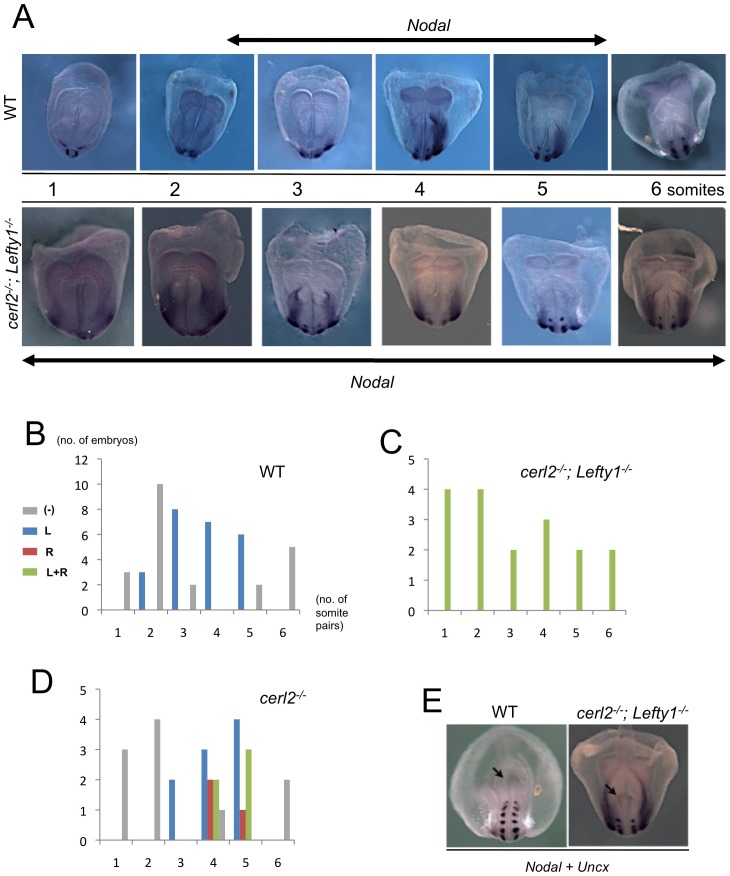
Displacement of antagonists at the node results in bilateral expression of *Nodal* in the LPM. (A) Whole-mount *in situ* hybridization for *Nodal* transcripts in wild-type, and *Cerl2^(−/−)^*; *Lefty1^(−/−)^* double-mutant mouse embryos during early somitogenesis. Expression of *Nodal* is left-sided in wild-type and always bilateral in *Cerl2^(−/−)^*; *Lefty1^(−/−)^*. The double arrows indicated the expression in embryos LPM during developmental stage, both in wild-type (3- to 5-somite stage) and double-mutant (0- to 6-somite-stage). (B–D) The relation between the sites of *Nodal* expression and developmental stages examined by whole-mount *in situ* hybridization. Gray bars, no expression detected; red bars, expression in the left LPM; blue bars, expression in the right-LPM; green bars, bilateral expression in LPM. (E) Comparison of the posterior view of a wild-type and *Cerl2^(−/−)^*; *Lefty1^(−/−)^* double-mutant at 6-somite stage. The double-mutant embryo still displays *Nodal* expression in the node as showed by the arrow.

The presence of active Nodal in the perinodal crown cells is essential for subsequent *Nodal* expression in the left-LPM [9]–[11]. Moreover, it has been reported that this dynamic expression is precisely timed, occurring between 2- to 5-somite stages in wild-type embryos [16], [22]. This can also be observed in Figure 4A. However, in the absence of *Cerl2*, half of the embryos show bilateral expression of *Nodal* in LPM, 40% unaffected left-sided expression, and 10% right-sided ectopic expression [14]. In addition, *Nodal* expression, is either left-sided, bilateral, or right-sided in the LPM of *Lefty1^(−/−)^* mutant embryos, depending on developmental stage [16]. It is important to note that the ectopic expression of *Nodal* in the right-LPM in these single mutants has distinct origins: in *Lefty1* ^(*−*/*−*)^ embryos, due to the absence of midline antagonism, an anterior ectopic expression of *Nodal* appears afterwards in the right-LPM by 4-somite stage due the diffusion of Nodal signals generated earlier (2-somite stage) on the left-LPM, while in *Cerl2* ^(*−*/*−*)^ single mutants the bilateral signal diffusion is in a posterior-to-anterior fashion via node that starts at 2-somite stage.

Here, to clarify these observations, we decided to analyze the expression of *Nodal* in the absence of both its node antagonists, Cerl2 and Lefty1. The *Cerl2* ^(*−*/*−*)^; *Lefty1* ^(*−*/*−*)^ double-mutant mice did not develop to term and miscarriage occurred around embryonic day 9.5 (data not shown). Whole-mount *in situ* hybridization performed in *Cerl2* ^(*−*/*−*)^; *Lefty1* ^(*−*/*−*)^ double-mutant embryos, showed that the expression of *Nodal* in the LPM was always bilateral in these mutants, and with a similar anterior-posterior extension in both LPMs (Fig. 4A,E). Interestingly, in *Cerl2* ^(*−*/*−*)^; *Lefty1* ^(*−*/*−*)^ double-mutant, the symmetric expression of *Nodal* on both LPM began much earlier, as soon as 1-somite stage, and is still present at 6-somite stage (Fig. 4A). Surprisingly, we also noticed that the expression of *Nodal* on the node disappears later in the *Cerl2* ^(*−*/*−*)^; *Lefty1* ^(*−*/*−*)^ double-mutant than in wild-type embryos (Fig. 4E). Overall, these results suggest that the accurate asymmetric and transient expression of *Nodal* in the left-LPM requires a coordinated activity of the Nodal antagonists at the level of the node. We hypothesize that Cerl2 is necessary to control the proper levels of active Nodal in the node during the first stages of early somitogenesis. Indeed, the activity of Nodal seems to be increased in *Cerl2* ^(*−*/*−*)^ embryos leading to ectopic and pronounced expression of *Lefty* genes in and on the posterior side of the node [22]. Nevertheless, although *Nodal* is expressed on the right side of the node, phosphorylated Smad2, a Nodal signaling readout, was never detected in this region [24]. Therefore, it is most likely that the level of active Nodal is not enough for the activation of its autoregulatory loop on the right side of the node due to the presence of Cerl2 protein. To further validate Cerl2 as an antagonist of Nodal activity, we used a luciferase assay utilizing an activin-responsive *A3-lux* reporter, which measures the activity of Nodal mediated by Smad proteins [25]. We have found that the cotransfection of both Nodal and Cerl2 expression constructs, in 293T cells, results in a strong inhibition of the signaling response when compared with Nodal transfection alone (Fig. S1).

**Figure 5:**
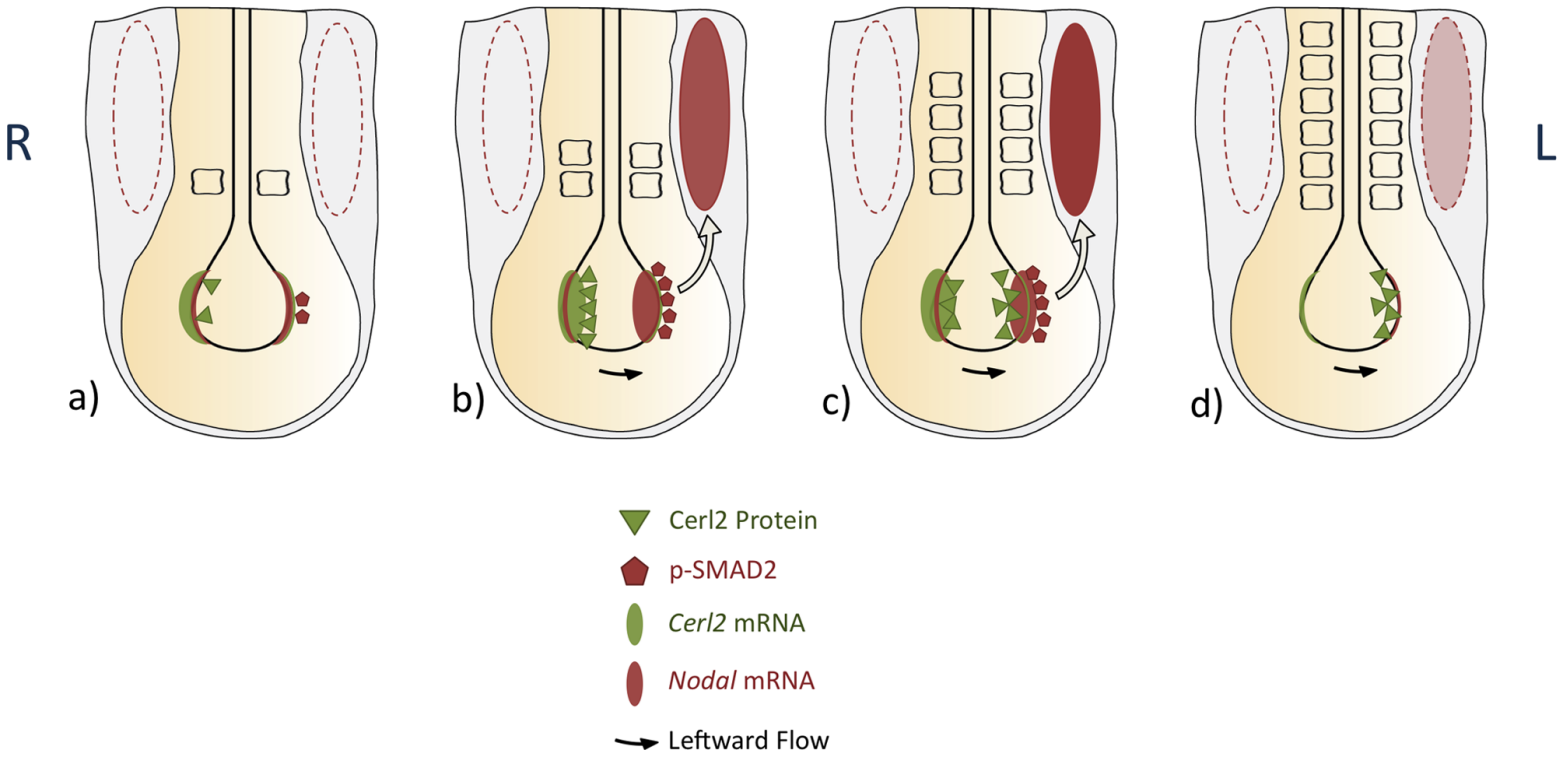
Sequential Nodal activity in left-right asymmetry at the mouse node. Summary of the symmetry-breaking events that occurs in the node during early somitogenesis. *Nodal* expression is represented in light red oval, and *Cerl2* expression in light green oval. The dynamic behavior of Cerl2 protein is illustrated in green triangles, and the readout of nodal signaling, pSmad2, is indicated in red pentagons. The asymmetric expression of *Nodal* in the left-LPM of mouse embryos is represented by the filled red oval. Dashed to thicker lines indicate increase in intensity. At 2-somite stage, Cerl2 protein (green triangles) localizes and prevents the activation of *Nodal* genetic circuitry on the right side of the embryo (dashed red oval). Later, due to nodal flow, Cerl2 right-to-left translocation shutdowns Nodal activity in the node and consequently affects the activity of Nodal in the LPM (dashed red oval). The arrows represent the nodal signal transfer across the node. a) 1-somite stage, b) 2-somite stage, c) 4-somite stage, d) 6-somite stage.

The right-to-left translocation of Cerl2 protein, at 2/3-somite stage, and its accumulation on the left side of the node until 5/6-somite stage, led us to propose that the left-sided presence of Cerl2 might decrease the local level of active Nodal at this stage. In fact, the phosphorylation of Smad2/3 starts to disappear at 5-somite stage in the node [24], exactly when Cerl2 strongly accumulates on the left crown cells, which is observed until the 6-somite stage (summarized in Fig. 5). In addition, it was reported that overexpression of *Coco* (frog *Cerl2* homolog) mRNA on the left-side of the node led to almost complete loss of L-LPM *Nodal* expression [26].

To support the idea that Cerl2 regulates the level of active Nodal in the node, we used the mathematical Self-Enhancement and Lateral-Inhibition (SELI) model constructed by Nakamura *et al.* (2006), which elegantly explains how the initial signal bias in the node is converted into robust asymmetry in the LPM, to simulate our experimental data. According to the model, when the initial level of Nodal signaling (*Ni*) is large enough (like in the L-LPM), the levels of Nodal and Lefty (1 and/or 2) will transiently increase, subsequently decrease, and finally disappear. However, when *Ni* is small (as in the R-LPM), the levels of Nodal and Lefty would converge to zero without increasing. Furthermore, when the *Ni* is large enough on both sides, the transient increase followed by decrease dynamics would appear on both sides, resulting in a bilateral phenotype [27]. The results showed that, when Cerl2 is not present, the initial Nodal signal (*Ni*) became persistent in the node and, thus, generate a continuous expression of *Nodal* in the LPM (the transient time window observed in the WT was lost) (Fig. S2). Taken together, the results of the mathematical model corroborate those observed in the *Cerl2* ^(*−*/*−*)^ and *Cerl2* ^(*−*/*−*)^; *Lefty1* ^(*−*/*−*)^ embryos in which Cerl2 appears to modulate the level of active Nodal in the node, which is reflected after in the left-LPM, in a precise time window.

The role of Cerl2 on the asymmetric activation of Nodal signaling cascade in the L-LPM seems to be conserved among vertebrates[6], [28]–[31]. However, to our knowledge, the studies performed so far only analyzed the effect of the flow in the level of *Cerl2* RNA expression (R>L) around the node, and subsequent left-sided release of *Nodal* repression. In frog, it was observed that the leftward flow represses *Coco (Cerl2)* on the left margin of the gastrocoel roof plate, up-regulating the co-expressed Nodal protein [29]. Moreover, the inhibition of flow by methylcellulose treatment or *dnah9*-MO injections causes a reduction of right-bias *Coco* (*Cerl2*) expression [29]. *Charon* (*Cerl2*), in fish, was reported to be asymmetrically expressed in Kupffer's vesicle, and this asymmetric expression seems to be regulated by KV flow since in flow-defective embryos, or with a disrupted KV, a symmetric expression is observed [32]. In mouse, the molecular mechanism underlying the conversion of the signal transmitted by nodal flow into R>L asymmetric expression of *Cerl2* was recently identified [33]. The *Cerl2* right-bias expression seems to be determined post-transcriptionally by the decay of *Cerl2* mRNA on the left side of the node due to both initial flow-derived signal and Wnt-mediated signaling [33]. Therefore, *Nodal* repression is released on the left side of the node, and the consequent L>R asymmetric pattern of Nodal activity is transmitted to the LPM, resulting in correct organ situs.

Here, we demonstrated that besides the R>L asymmetric RNA expression of *Cerl2*, the right-to-left dynamic localization of Cerl2 protein is also important to control the level of active Nodal at the highly sensitive system that is the mouse node. In the absence of both inhibitors, in *Cerl2^(−/−)^*; *Lefty1^(−/−)^* embryos, Nodal increases and prolongs its RNA self-expression not only on the node but also in the LPM (Fig. S3). More, we showed that the Cerl2 right-to-left displacement is also nodal flow-dependent. Therefore, we propose Cerl2 as a key molecule in the mechanism of laterality determination, able to comprise the two principal flow action models (two-cilia and transported determinant morphogen). From EHF until 2-somite stage, *Cerl2* right-sided asymmetric RNA expression seems to be regulated by a signal transmitted by the local nodal flow (two-cilia model). This leads to an accumulation of Cerl2 protein on the right side of the node that prevents the activation of Nodal cascade on the right-LPM. At 2/3-somite stage, in its right-to-left localization, Cerl2 protein acts as a morphogen determinant in L-R asymmetry taking advantage of the maximum global nodal flow (transported determinant molecule model). The presence of Cerl2 protein on the left side represses the activity of Nodal in the node, and by the 6-somite stage the transfer of laterality information to the left side of the embryo ceases. Furthermore, this explains the precise time window in which *Nodal* is expressed in the left-lateral plate mesoderm.

## Supporting Information

Figure S1 **Inhibition of Nodal activity by Cerl2.** Mixtures containing 0.5 µg of the indicated plasmids, 50 ng of the luciferase reporter plasmid, 20 ng of CMV-β-Gal plasmid, and various amounts of pCS2+ vector to maintain a constant amount of total DNA were used to transfect 293T cells. Luciferase activity was measured 48 h after transfection, and the activities were normalized to β-galactosidase control (RLU). The results represent the average of triplicates of three independent experiments.

Figure S2 **Mathematical model of LR patterning in mouse embryos.** Mathematical simulation of *Nodal* and *Lefty (1 and/or 2)* expression in the wild-type (WT), *Cerl2^(−/−)^* single mutant, and *Cerl2^(−/−)^; Lefty1^(−/−)^* double-mutant embryos, showing that Cerl2 controls the initial signal received by the LPM. In its absence, the initial signal became persistent and the expression of *Nodal* in the LPM uninterrupted.

Figure S3 **Propagation of the nodal pathway from the node to the lateral plate mesoderm in wild-type and** ***Cerl2^(−/−)^; Lefty1^(−/−)^*** **double-mutant embryos.** In the absence of Nodal antagonism in the node, by loss of both Cerl2 and Lefty1, the expression of *Nodal* in LPM became always bilateral, earlier and wider than expected. This is, most probably, due to a combination of both anterior-posterior and posterior-anterior propagation of the signal observed in the single *Lefty1^(−/−)^* and single *Cerl2^(−/−)^* mutants, respectively. *Cerl2* expression (green cells), *Nodal* expression (red cells), *Lefty1* expression (blue circles), and Cerl2 protein (green triangles) are denoted. The arrows represent the nodal signal transfer across the embryo.

## References

[1] Hamada H, Meno C, Watanabe D, Saijoh Y (2002) Establishment of the vertebrate left-right asymetry. Nature Reviews Genetics 3: 103–113. 10.1038/nrg732 11836504

[2] Raya A, Belmonte JC (2006) Left-right asymmetry in the vertebrate embryo: from early information to higher-level integration. Nature Reviews Genetics 7: 283–293. 10.1038/nrg1830 16543932

[3] Shen MM (2007) Nodal signaling: developmental roles and regulation. Development 134: 1023–1034. 17287255 10.1242/dev.000166

[4] Ramsdell AF (2005) Left-right asymmetry and cogenital cardiac defects: getting to the heart of the matter in vertebrate left-right axis determination. Developmental Biology 288: 1–20. 16289136 10.1016/j.ydbio.2005.07.038

[5] Brueckner M (2007) Heterotaxia, congenital heart disease, and primary ciliary dyskinesia. Circulation 115: 2793–2795. 17548739 10.1161/CIRCULATIONAHA.107.699256

[6] Hirokawa N, Tanaka Y, Okada Y, Takeda S (2006) Nodal flow and the generation of the left-right asymmetry. Cell 125: 35–45. 10.1016/j.cell.2006.03.002 16615888

[7] Capdevila J, Vogan KJ, Tabin CJ, Izpisua-Belmonte JC (2000) Mechanisms of left-right determination in vertebrates. Cell 101: 9–21. 10778851 10.1016/S0092-8674(00)80619-4

[8] Wright C V (2001) Mechanisms of the left-right asymmetry: What's right and what's left? Developmental Cell 1: 179–186. 11702778 10.1016/s1534-5807(01)00036-3

[9] Brennan J, Norris DP, Robertson EJ (2002) Nodal activity in the node governs left-right asymmetry. Genes & Development 16: 2339–2344. 12231623 10.1101/gad.1016202 PMC187443

[10] Saijoh Y, Oki S, Hamada H (2003) Left-right patterning of the mouse lateral plate requires nodal produced in the node. Developmental Biology 256: 161–173. 10.1016/s0012-1606(02)00121-5 12654299

[11] Oki S, Hashimoto R, Okui Y, Shen MM, Mekada E, et al (2007) Sulfated glycosaminoglycans are necessary for Nodal signal transmission from the node to the left lateral plate in the mouse embryo. Development 134: 3893–3904. 17913787 10.1242/dev.009464

[12] Shiratori H, Hamada H (2006) The left-right axis in the mouse: from origen to morphology. Development 133: 2095–2104. 16672339 10.1242/dev.02384

[13] Belo JA, Silva AC, Borges AC, Filipe M, Bento M, et al (2009) Generating asymmetries in the early vertebrate embryo: the role of te Cerberus-like family. International Journal of Developmental Biology 53: 1399–1407. 19247954 10.1387/ijdb.072297jb

[14] Marques S, Borges AC, Silva AC, Freitas S, Cordenonsi M, et al (2004) The activity of the Nodal antagonist Cerl-2 in the mouse node is required for correct L/R body axis. Genes & Development 18: 2342–2347. 15466485 10.1101/gad.306504 PMC522983

[15] Okada Y, Takeda S, Tanaka Y, Belmonte JC, Hirokawa N (2005) Mechanism of nodal flow: a conserved symmetry breaking event in left-right axis determination. Cell 121: 633–644. 15907475 10.1016/j.cell.2005.04.008

[16] Meno C, Shimono A, Saijoh Y, Yashiro K, Mochida K, et al (1998) Lefty-1 is required for left-right determination as regulator of the lefty-2 and nodal. Cell 94: 287–297. 9708731 10.1016/s0092-8674(00)81472-5

[17] Belo JA, Bouwmeester T, Leyns L, Ketersz N, Gallo M, et al (1997) Cerberus-like is a secreted factor with neutralizing activity expressed in the anterior primitive endoderm of the mouse gastrula. Mechanisms of Development 68: 45–57. 9431803 10.1016/s0925-4773(97)00125-1

[18] Yan Y, Liu J, Luo Y, Chaosu E, Haltiwanger RS, et al (2002) Dual Roles of Cripto as a Ligand and Coreceptor in the Nodal Signaling Pathway. Molecular and Cellular Biology 22: 4439–4449. 12052855 10.1128/MCB.22.13.4439-4449.2002 PMC133918

[19] Colognato H, Yurchenco PD (2000) Form and function: the laminin family of the heterotrimers. Developmental Dynamics 218: 213–234. 10842354 10.1002/(SICI)1097-0177(200006)218:2<213::AID-DVDY1>3.0.CO;2-R

[20] Okada Y, Nonaka S, Tanaka Y, Saijoh Y, Hamada H, et al (1999) Abnormal nodal flow precedes situs inversus in the in and inv mice. Molecular Cell 4: 459–468. 10549278 10.1016/s1097-2765(00)80197-5

[21] Shinohara K, Kawasumi A, Takamatsu A, Yoshiba S, Botilde Y, et al (2012) Two rotating cilia in the node cavity are sufficient to break left-right symmetry in the mouse embryo. Nature Communications 3: 622. 10.1038/ncomms1624 22233632

[22] Oki S, Kitajima K, Marques S, Belo JA, Yokoyama T, et al (2009) Reversal of left-right asymmetry induced by aberrant Nodal signaling in the node of mouse embryos. Development 136: 3917–3925. 19906859 10.1242/dev.039305

[23] Larkins CE, Long AB, Caspary T (2012) Defective Nodal and Cerl2 expression in the Arl13bhnn mutant node underlie its heterotaxia. Developmental Biology 367: 15–24. 22554696 10.1016/j.ydbio.2012.04.011

[24] Kawasumi A, Nakamura T, Iwai N, Yashiro K, Saijoh Y, et al (2011) Left-right asymmetry in the level of active Nodal protein produced in the node is translated into left-right asymmetry in the lateral plate of the mouse embryos. Developmental Biology 353: 321–330. 21419113 10.1016/j.ydbio.2011.03.009 PMC4134472

[25] Liu F, Pouponnot C, Massagué J (1997) Dual role of the Smad4/DPC4 tumor suppressor in the TGFβ signals. Genes & Development 11: 3157–3167. 9389648 10.1101/gad.11.23.3157 PMC316747

[26] Vonica A, Brivanlou AH (2007) The left-right axis is regulated by the interplay of Coco, Xnr1 and derrière in Xenopus embryos. Developmental Biology 303: 281–294. 17239842 10.1016/j.ydbio.2006.09.039

[27] Nakamura T, Mine N, Nakaguchi E, Mochizuchi A, Yamamoto M, et al (2006) Generation of robust left-right asymmetry in the mouse embryo requires a Self-Enhancement and Lateral-Inhibition system. Developmental Cell 11: 495–504. 17011489 10.1016/j.devcel.2006.08.002

[28] Tavares AT, Andrade S, Silva AC, Belo JA (2007) Cerberus is a feedback inhibitor of Nodal asymmetric signaling in the chick embryo. Development 134: 2051–2060. 17507406 10.1242/dev.000901

[29] Schweickert A, Vick P, Getwan M, Weber T, Schneider I, et al (2010) The nodal inhibitor Coco is a critical target of leftward flow in Xenopus. Current Biology 20: 738–743. 20381352 10.1016/j.cub.2010.02.061

[30] Essner JJ, Amack JD, Nyholm MK, Harris EB, Yost HJ (2005) Kupffer's vesicle is a ciliated organ of asymmetry in the zebrafish embryo that initiates left-right development of the brain, heart and gut. Development 132: 1247–1260. 15716348 10.1242/dev.01663

[31] Nonaka S, Tanaka Y, Okada Y, Takeda S, Harada A, et al (1998) Randomization of the left-right asymmetry due to loss of nodal cilia generating leftward flow of extraembryonic fluid in mice lacking KIF3B motor protein. Cell 95: 829–837. 9865700 10.1016/s0092-8674(00)81705-5

[32] Hojo M, Takashima S, Kobayashi D, Sumeragi A, Shimada A, et al (2007) Right-elevated expression of charon is regulated by fluid flow in medaka Kupffer's vesicle. Development, Growth & Differentiation 49: 395–405. 10.1111/j.1440-169X.2007.00937.x 17547649

[33] Nakamura T, Saito D, Kawasumi A, Shinohara K, Takaoka K, et al (2012) Fluid Flow and Interlinked Feedback Loops Establish Left-Right Asymmetric Decay of Cerl2 mRNA in the Mouse Embryo. Nature Communications 3: 1322. 10.1038/ncomms2319 23271656

