# Supplementary figures and images for: The Dynamic Right-to-Left Translocation of Cerl2 Is Involved in the Regulation and Termination of Nodal Activity in the Mouse Node
Source: PLoS One. 2013 Mar 27;8(3):e60406. doi: 10.1371/journal.pone.0060406 (PMC3609817; doi:10.1371/journal.pone.0060406)

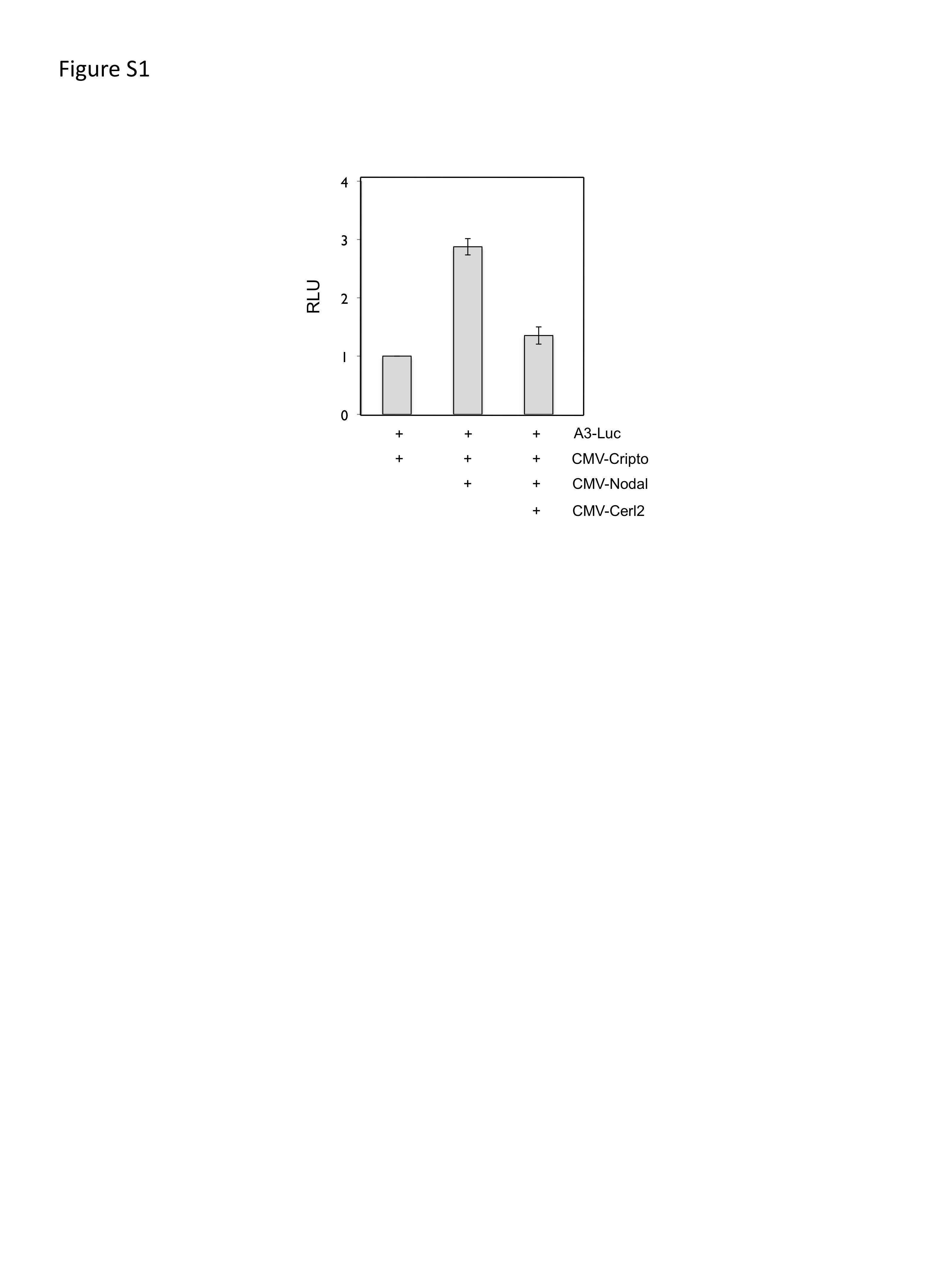

Supplement: Figure S1 — Inhibition of Nodal activity by Cerl2. Mixtures containing 0.5 µg of the indicated plasmids, 50 ng of the luciferase reporter plasmid, 20 ng of CMV-β-Gal plasmid, and various amounts of pCS2+ vector to maintain a constant amount of total DNA were used to transfect 293T cells. Luciferase activity was measured 48 h after transfection, and the activities were normalized to β-galactosidase control (RLU). The results represent the average of triplicates of three independent experiments. [file pone.0060406.s001.tif]

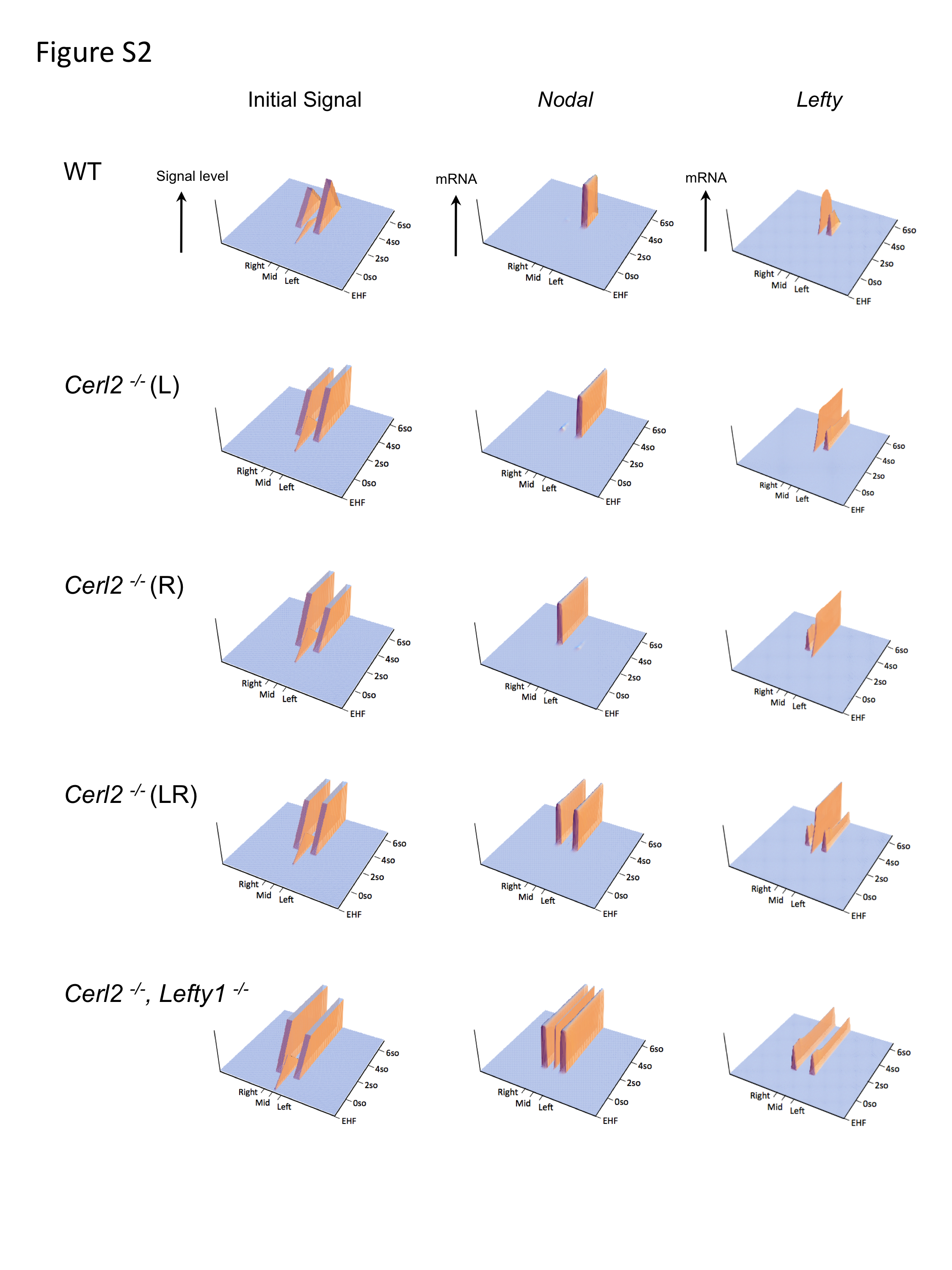

Supplement: Figure S2 — Mathematical model of LR patterning in mouse embryos. Mathematical simulation of Nodal and Lefty (1 and/or 2) expression in the wild-type (WT), Cerl2(−/−) single mutant, and Cerl2(−/−); Lefty1(−/−) double-mutant embryos, showing that Cerl2 controls the initial signal received by the LPM. In its absence, the initial signal became persistent and the expression of Nodal in the LPM uninterrupted. [file pone.0060406.s002.tif]

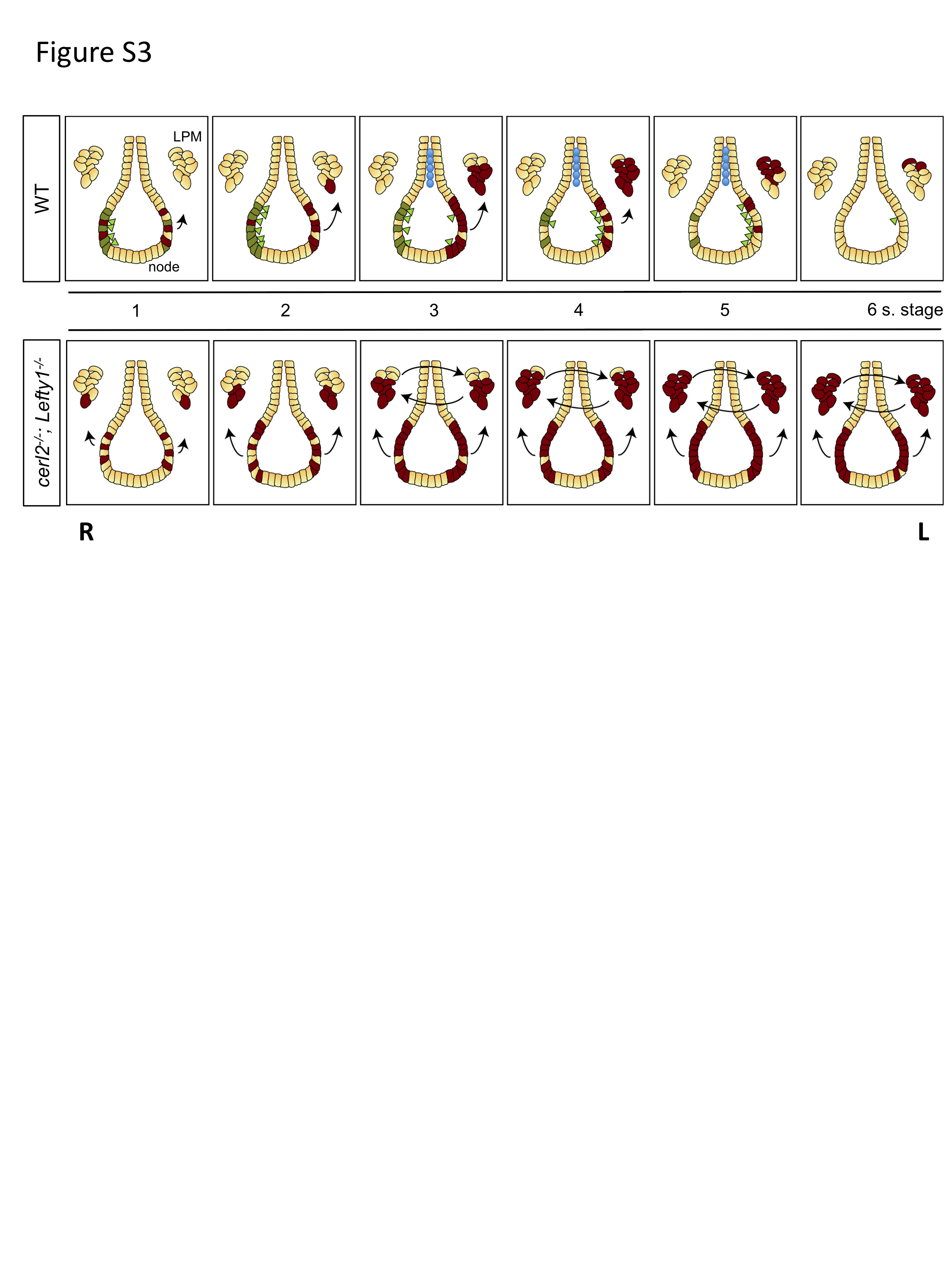

Supplement: Figure S3 — Propagation of the nodal pathway from the node to the lateral plate mesoderm in wild-type and Cerl2(−/−); Lefty1(−/−) double-mutant embryos. In the absence of Nodal antagonism in the node, by loss of both Cerl2 and Lefty1, the expression of Nodal in LPM became always bilateral, earlier and wider than expected. This is, most probably, due to a combination of both anterior-posterior and posterior-anterior propagation of the signal observed in the single Lefty1(−/−) and single Cerl2(−/−) mutants, respectively. Cerl2 expression (green cells), Nodal expression (red cells), Lefty1 expression (blue circles), and Cerl2 protein (green triangles) are denoted. The arrows represent the nodal signal transfer across the embryo. [file pone.0060406.s003.tif]
